# Identity construction among second-generation Chinese in Italy: a perspective on the symbolic function of language

**DOI:** 10.3389/fpsyg.2026.1826033

**Published:** 2026-07-27

**Authors:** Tu Haiqiang, Guo Jun, Wang Jia Yi

**Affiliations:** School of International Educational, Wenzhou University, Wenzhou, China

**Keywords:** identity construction, language use, literacy proficiency, second-generation Chinese in Italy, symbolic function

## Abstract

As a distinct demographic navigating a cross-cultural landscape, second-generation Chinese immigrants in Italy exhibit patterns of language use that serve as crucial indicators of their identity construction. Based on a quantitative analysis of scales measuring identity, language proficiency, and linguistic behavior, this study identifies a structural paradox within this group characterized by the coexistence of “low literacy proficiency” and “strong ethnic identity.” The findings suggest that the function of the Chinese language for these individuals is shifting from a purely functional skill to a symbolic marker, acting as a primary vehicle for cultural cohesion. This symbolic usage is predominantly evident in family interactions, identity performance within peer groups, and contexts of emotional expression. The study concludes that this symbolic mode of language use constitutes a mechanism for identity maintenance that operates independently of actual literacy skills, thereby offering a novel interpretive framework for understanding heritage language acquisition and cross-cultural identity formation.

## 1 Introduction

The interplay between language and identity has long constituted a focal point in sociolinguistics and intercultural studies (Labov, 1966; Rajagopalan, 2016; Tajfel, 1978; Adler, 1927; Ang, 2001; Baker, 1992; Edwards, 1985; Ferrer and Sankoff, 2003; Giles and Johnson, 1987; Lay and Verkuyten, 1999; Smolicz, 1981; Wang and Yan, 2011; Zhang, 2015; Zhang, 2020; Zhang et al., 2021; Erikson, 1950). Early scholarship predominantly emphasized the communicative and cultural-bearing functions of language, positing that linguistic proficiency serves as the fundamental basis for cultural comprehension, emotional belonging, and the construction of ethnic identity (Cao and Yang, 2014; Zhou, 2016). However, as research into heritage languages (Guo, 2015) has deepened, a growing body of scholarship indicates that a decline in linguistic proficiency does not inevitably precipitate a weakening of identity or cultural affiliation. This detachment is particularly evident within overseas Chinese communities, where the social symbolic attributes of language are becoming increasingly salient (Wang, 2004, 2006; Zhu and Wang, 2012).

According to Bourdieu (1991), a seminal figure in the theory of symbolic functions, characterizes language as a form of “symbolic capital.” He argues that the value of language derives not merely from its technical utility but from the recognition and legitimacy conferred upon it within a specific social context. In this view, language use frequently transcends competence, intertwining closely with individual identity, power dynamics, and social positioning. Further elaborating on this, Le Page and Tabouret-Keller (1985) proposed the theory of “Acts of Identity,” suggesting that individuals employ specific linguistic forms to achieve self-positioning or group affiliation. Consequently, language functions as a vehicle for identity expression and negotiation—a symbolic significance that, in numerous scenarios, supersedes its pragmatic communicative function (He, 2006; Stratilaki, 2012).

In studies concerning the Chinese diaspora, this symbolic function has garnered increasing attention. Empirical evidence suggests that while many second- or third-generation Chinese immigrants may struggle with fluency, they consciously elect to use Chinese during family interactions, festivals, or peer exchanges to articulate a sense of belonging or a specific emotional stance (Guo, 2017; Zhou, 2014;Bai et al., 2014; Bao and Li, 2023; Shen, 2017; Wang and Yan, 2011; Zhang, 2015, 2020; Zhang et al., 2021). To a certain extent, the role of language within these groups has transcended daily communication, assuming the function of a cultural symbol and identity marker. Concurrently, some heritage language studies observe that in multilingual environments, while literacy skills are often the most arduous to maintain, identity can nonetheless be reinforced through symbolic usage and specific contextual engagement (Guo, 2023).

With the intergenerational transition in the Italian Chinese immigrant community, the “new generation” raised in a multilingual environment has gradually become the demographic core. Traditional heritage language assumptions often presume that “competence determines identity.” However, recent European diaspora studies (e.g., Paciocco, 2018; Paciocco, 2021) and long-term empirical observations show this view is overly absolute; many Italian-Chinese youth facing significant literacy challenges still exhibit a strong sense of ethnic belonging. Although the symbolic role of language is widely acknowledged in macro-diaspora studies, its micro-level operational mechanisms in specific multilingual ecologies remain underexplored. Compared to North American contexts, there is a lack of empirical research using daily interaction data in the Italian Chinese community. Furthermore, existing literature rarely addresses the natural “literacy gap” inherent to the Chinese logographic system, nor how this proficiency differentiation inadvertently fosters compensatory identity mechanisms at the affective level.

To address these gaps and avoid circular reasoning, this study establishes a strict operational distinction between the “instrumental” and “symbolic” functions of language. The instrumental function is quantitatively evaluated through objective listening, speaking, reading, and writing proficiency indicators. Conversely, the symbolic function is defined specifically at the affective and indexical levels as a performative marker of ethnic identity. Methodologically, this study separates respondents’ subjective affective attitudes from their objective proficiency levels. This is complemented by semi-structured interviews to examine micro-level ritualistic use of Chinese, stance-taking, and code-switching behaviors within family and community networks.

Based on this rigorous theoretical framework, this study attempts to answer: Why does the weakening of literacy abilities not diminish identity? How is the symbolic function of Chinese formed and enacted in daily interactions? To what extent does it constitute an alternative pathway for identity maintenance? In summary, by analyzing the micro-mechanisms of language practices among the new generation of Chinese immigrants in Italy, this study aims to further reveal the underlying logic of weakened competence alongside persistent identity.

## 2 Method

### 2.1 Participants

The participants of this survey comprised 189 members of the new generation of Chinese immigrants attending Chinese language schools in Italy. The study specifically targets Chinese adolescents who were either born in Italy or immigrated with their parents during the preschool stage, and who have completed their primary compulsory education in the local context. This demographic profile is considered to possess typical “heritage language learner characteristics” (Cao, 2014).

The respondents for this questionnaire study were drawn from two large-scale Chinese heritage schools located in key cities with concentrated Chinese populations in central Italy, with the sample spanning from elementary to high school. These institutions implement comprehensive and well-established Chinese curriculum systems, requiring students to pass rigorous final examinations to advance to the next grade. Consequently, utilizing students from these two schools as the research sample allows their grade levels to serve as an objective indicator of their actual Chinese proficiency, thereby validating the measurement approach. Based on this criterion, the sample was stratified into a high proficiency group and a low proficiency group during data collection to facilitate a comparative analysis of language proficiency and identity characteristics across different stages. Specifically, elementary school students were categorized into the low proficiency group, while middle and high school students were assigned to the high proficiency group.

### 2.2 Measures

Through a questionnaire survey, this study systematically investigates the language proficiency structure, level of identity, and behavioral characteristics related to the symbolic use of Chinese among the new generation of Chinese immigrants. A combination of quantitative and qualitative analytical methods is adopted.

A quantitative approach was adopted to analyze data across the core component scales. Module 1 is the Cultural Identity Module, which draws upon previous research regarding overseas Chinese identity (He, 2023; Xu, 2022). This module consists of 17 items, all measured on a 5-point Likert scale (ranging from 1 = Strongly disagree/Very unwilling to 5 = Strongly agree/Very willing). These 17 items stratified identity into four dimensions—ethnic identity, national identity, cultural identity, and value identity—constituting the core Quantitative Identity Scale utilized for all statistical modeling and correlation analyses.

Module 2 evaluates Language Use and Attitudes. To ensure the construct validity and transparency of the measurement instruments, this study rigorously operationalized the questionnaire indicators. Based on established heritage language attitude scales, the questionnaire was localized to fit the multilingual ecology of the Chinese community in Italy and underwent a rigorous forward–backward translation procedure between Chinese and Italian to ensure conceptual equivalence. This module consists of 13 continuous 5-point Likert items measuring two core variables: Objective Proficiency (8 items, covering listening [N1], speaking [N2–N4, assessing fluency, accuracy, and complexity], reading [N5], and writing [N6–N8, covering character, sentence, and discourse levels]) and Symbolic Attitudes (5 items [T1–T5], divided into cognitive and affective dimensions).

Furthermore, an additional Language Behavior component measures the frequency of using Chinese versus Italian across various settings (family, community, school, and public spaces). To avoid conflating language use driven by communicative necessity (e.g., interacting with elders) with symbolic behavior, the structural modeling strictly employed the meaning-oriented indicators of T3–T5 to capture the respondents’ affective stances. The structure and operationalization of these core continuous variables are detailed in Table 1.

**Table 1:** Structure and operationalization of the core measurement variables.

Main Module	Sub-dimension	No. of Likert items	Example item
Module 1: Identity scale (17 items)	Ethnic identity	4	I would feel proud if any Chinese people would receive honors internationally
	National identity	4	I would be very angry if someone said bad things about China or Chinese people
	Cultural identity	4	I believe that Chinese culture is vast and profound, with a long and rich history
	Value identity	5	I believe that traditional Chinese family values are very necessary
Module 2: Language scale (13 items)	Objective proficiency (N1–N8)	8	When communicating with people in Chinese, I am able to accurately express my meaning
	Symbolic attitudes (T1–T5)	5	Speaking Chinese makes me very friendly (with a sense of belonging)

The quantitative structural and correlation analyses utilized these 30 core items evaluated on a 5-point Likert scale. See Appendix A for the full instrument.

During the qualitative phase, interviews serve as an essential research method to provide an in-depth understanding of the language use experiences and identity construction processes of the new generation of Chinese immigrants in Italy across different contexts. This study adopts a semi-structured interview format to ensure both flexibility and depth. The interviewees consist of two groups: students of the new generation of Chinese immigrants and Chinese language teachers. Through these semi-structured qualitative interviews, initial questions are posed to the respondents, followed by probing questions based on their answers, in order to further explore their language use motivations, explicitly asking respondents to explain their underlying motivations for choosing Chinese, code-switching phenomena, and shifts in identity.

### 2.3 Data analysis

To guarantee the methodological rigor of the questionnaire and the reliability of the analytical results, this study conducted reliability and validity tests. The results indicate that the Cronbach’s *α* coefficient for each scale exceeds 0.80, and the KMO values are all above 0.75. On this basis, SPSS 26.0 was employed to analyze the language proficiency structure and identity characteristics of the new generation of Chinese immigrants in Italy. Through inter-group difference testing, the study empirically dissects the correlation between the two, aiming to unveil the evolutionary trajectory of the function of language within the identity construction of this demographic.

## 3 Results

### 3.1 Structure of language proficiency

**Figure 1:**
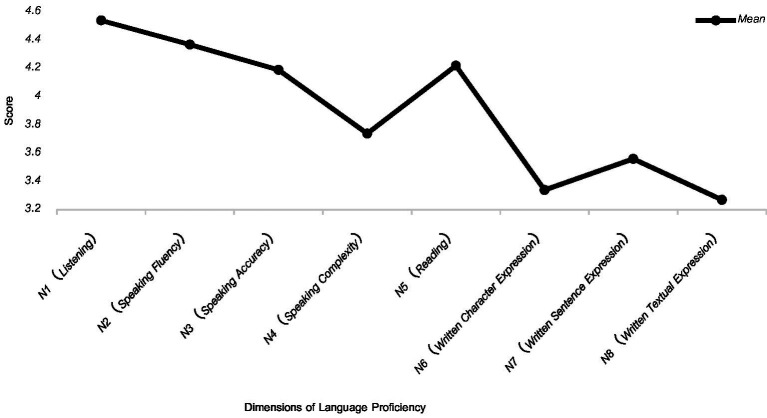
Overall language proficiency structure of participant. Language proficiency was measured on a 5-point Likert scale ranging from 1 (very poor) to 5 (excellent).

The overall distribution in Figure 1 demonstrates a distinct stratification of skills in the language proficiency of the new generation. Benefiting from the natural immersive environment provided by families and the Chinese community, the participants’ scores in listening comprehension (*M* = 4.54) and speaking fluency (*M* = 4.37) remain at a high level. However, as skill requirements deepen, particularly within the domain of written language, scores exhibit a precipitous decline. It is evident that the participants’ reading (N5) capabilities generally lag behind their oral-aural skills, while text writing (N8) constitutes the lowest scoring item across the entire sample. This structural configuration is largely consistent with previous findings in heritage language contexts regarding the “ease of maintaining oral skills versus the difficulty of maintaining literacy” (Cao, 2014), providing a contextual basis for the subsequent discussion on the potential symbolic turn of language functions.

Notably, within the generally low-scoring written expression dimension, the score for “written sentence expression” (N7, *M* = 3.56) is marginally higher than those for “character writing” (N6, *M* = 3.34) and “textual expression” (N8, *M* = 3.27). This micro-level discrepancy stems from a positive transfer mechanism from oral capabilities: since Chinese syntax aligns highly with oral expression, participants can leverage their fluent oral sense to support sentence construction. Conversely, character writing relies on mechanical memory of logograms (orthographic awareness), while textual expression depends on systematic logical training, neither of which can be acquired solely through oral practice. Consequently, the new generation falls into a dilemma of “functional semi-literacy”—capable of speaking in sentences but struggling to recall characters or compose texts—resulting in an objective rupture in their instrumental literacy skills.

**Figure 2:**
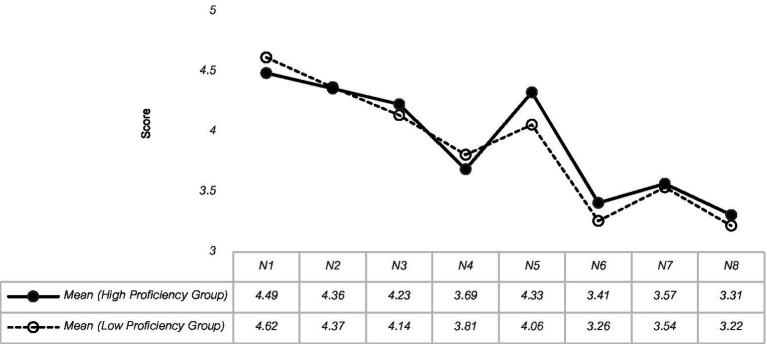
Language proficiency structure of participants at different levels. Participants were categorized into high-proficiency and low-proficiency groups based on their comprehensive scores.

To investigate capability differences across distinct proficiency levels, the study grouped participants based on comprehensive scores. Tests reveal that the differences between the high-literacy group and the low-literacy group in listening (N1) and speaking (N2–N4) dimensions are not significant; however, marked divergence appears in character recognition, text reading, and written expression. This suggests that while aural-oral skills remain relatively uniform across students of varying Chinese proficiency, reading ability serves as the critical dimension distinguishing internal differences in language competence among the new generation. See Figure 2.

The data in Figure 2 essentially reflects a polarization in the language proficiency of respondents. On one hand, oral and listening skills exhibit a “ceiling effect.” Since the participants grew up in Chinese families, their foundational aural-oral abilities have reached a high plateau, resulting in minimal inter-group variance and making it difficult to discern gaps. On the other hand, writing skills demonstrate a “floor effect.” Due to the inherent structural complexity of Chinese characters and the difficulty of learning them, character acquisition requires sustained and substantial investment of time and energy. This renders the character production output of the vast majority of participants universally weak, clustering at the lower end of the scale, which similarly fails to form an effective distinction. Only reading ability occupies the middle ground; it does not require the arduous retrieval of character forms as writing does, yet it demands more vocabulary accumulation and active cognition than mere listening. Thus, reading level authentically reflects the degree of personal investment beyond natural family acquisition (such as the cultivation of extracurricular reading interests), thereby becoming the decisive watershed for distinguishing language proficiency levels.

### 3.2 Identity characteristics

Despite the pervasive decline in literacy proficiency, data concerning identity manifests a stability that appears independent of technical skills. Statistical results indicate that participants universally achieved elevated scores on the identity scale, accompanied by a narrow standard deviation. This attests to a high degree of internal consistency regarding the affirmation of Chinese ethnicity within this demographic.

**Figure 3:**
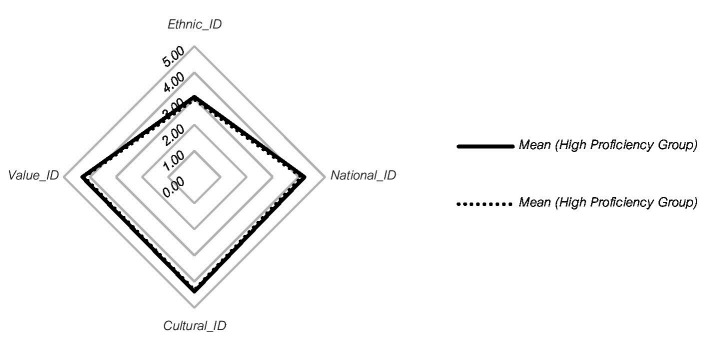
Identity characteristics of participants at different levels. Identity scores represent the strength of identification with each dimension (Ethnic, National, Cultural, Value), rated on a scale from 1 (strongly disagree) to 5 (strongly agree).

Further comparative analysis via Figure 3 reveals that even the “low-proficiency group,” characterized by the weakest literacy skills, exhibited no statistically significant divergence from the “high-proficiency group” regarding national, value, and cultural identity. This phenomenon of “consistency in identity despite disparity in capability” unveils the relative autonomy of the generative mechanisms underlying identity versus those of linguistic competence.

This elevated level of identification is largely attributable to the “oralization” and “lifestyle-orientation” of cultural transmission paths. The cultural and value identities examined in this study (e.g., traditional customs, family ethics) rely predominantly on verbal interaction and quotidian practices within the household (e.g., festival rituals, dietary habits) rather than necessitating advanced character decoding skills. Consequently, even amidst obstacles in written language, the new generation can bypass the “literacy threshold” through immersion in family life, thereby acquiring tangible experience of and identification with Chinese culture.

Furthermore, Figure 3 reflects a “dual differentiation” between emotional belonging and instrumental skills among the new generation. Ethnic and national identities are intrinsically deep-seated emotional attachments and psychological sets, possessing strong nativist characteristics; in contrast, literacy constitutes an instrumental skill demanding prolonged training. The developmental trajectories of these two domains are not entirely synchronous. It is plausible that certain members of the new generation, cognizant of their linguistic deficits, tend to employ a “psychological compensation mechanism” (King et al., 2008) By reinforcing identity belonging at the psychological level to compensate for imperfections in linguistic competence, they succeed in sustaining a positive self-confirmation of identity.

### 3.3 Correlation between literacy and identity

A Pearson correlation analysis was conducted to examine the relationship between language proficiency and identity (see Table 2). The results reveal a significant positive correlation between the total language proficiency score and the total identity score (*r* = 0.422, *p* < 0.001). This suggests that, in terms of the overall trend, superior language proficiency indeed facilitates the construction of a stronger identity. Functioning as a form of “cultural capital,” linguistic skills afford individuals profounder cultural experiences and confidence, thereby generating a “value-added effect” on identity.

**Table 2:** Correlation analysis between literacy proficiency and identity.

Variables	Literacy proficiency	Identity	*M*	SD
Literacy proficiency	1	0.422^**^	3.60	0.57
Identity	0.422^**^	1	15.76	1.76

*M*, Mean; SD, Standard Deviation. Literacy proficiency and identity scores are aggregate measures. ** indicate *p*<0.01.

Concurrently, the magnitude of the correlation coefficient (*r* = 0.422) indicates that literacy proficiency does not fully determine identity. When calculated via the coefficient of determination (*r*^2^), literacy proficiency accounts for approximately 19% of the variance in identity, whereas over 80% of identity intensity is determined by factors extrinsic to linguistic skills. Therefore, while language proficiency is a significant variable regarding identity, it is not a determinant one. In other words, while identity can be reinforced to a certain extent through literacy, high literacy is not a prerequisite condition. For the vast majority of the new generation, the foundation of their identity remains solid even in the absence of advanced literacy. This provides empirical support for our subsequent exploration of the “symbolic function of language”—positing that language may function within identity maintenance more as an affective symbol than as a technical tool.

### 3.4 Symbolic function of language

**Figure 4:**
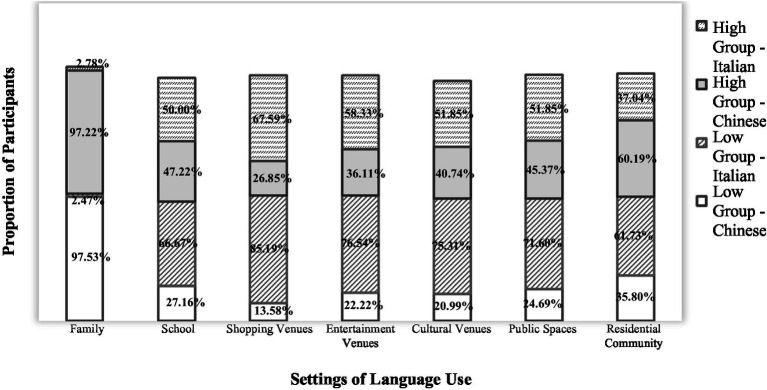
Frequency of language use by participants of different levels across various settings.

**Figure 5:**
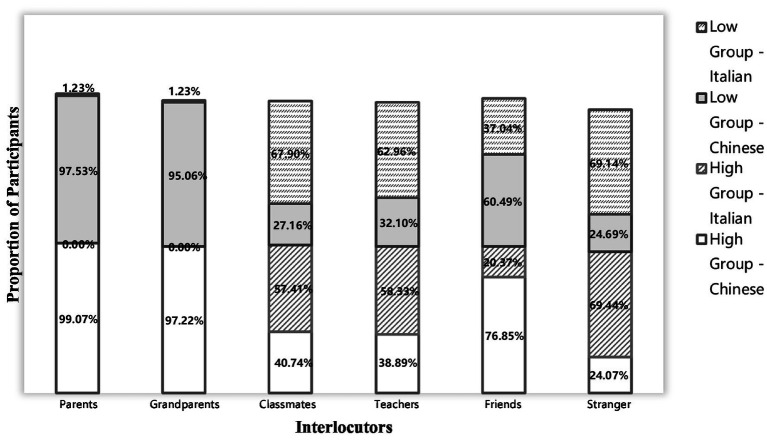
Frequency of language use by participants of different levels. Data represents the mean frequency of Chinese language use in various settings (or with various interlocutors). Higher scores indicate a greater frequency of using Chinese over Italian.

To gain a more concrete understanding of the symbolic function of language, this study further examined the proportion of participants at different proficiency levels who use Chinese versus Italian across various settings and with different interlocutors (see Figures 4, 5).

Data from Figures 4, 5 reveal that in interactions within the family and with elders, Chinese retains its fundamental communicative function. Even students with weaker literacy skills exhibit a propensity to use Chinese for communication. In these instances, the deployment of Chinese is driven more by the imperatives of etiquette, habit, and affective expression. Conversely, in peer interactions, Italian assumes a dominant position, while the use of Chinese manifests distinct characteristics of “fragmentation” and “symbolization.” Some students mentioned in their self-narratives that “speaking a few sentences of Chinese lets others know I am Chinese,” or that they use Chinese “to discuss secrets with friends in specific situations so others cannot understand.” These statements corroborate indirectly that the new generation tends to employ Chinese selectively during moments of specific emotional expression, identity assertion, or intra-group “secret communication.” At this juncture, Chinese is no longer utilized to pursue the efficiency of information transmission but is displayed as a “credential of identity.” This usage pattern indicates that within the new generation, Chinese has gradually divested itself of the attribute of a mere communicative tool, evolving instead into a symbolic cultural marker that demarcates ethnic boundaries and confirms “in-group” membership.

## 4 Discussion

### 4.1 Reconfiguration of linguistic value

Bourdieu’s (1991) theory of linguistic capital posits that linguistic competence is not merely a technical ability but a form of capital, the value of which depends on the profit of distinction it can yield within a specific field. This framework offers a cogent explanation for the structural phenomenon of “high identity coexistence with low literacy” observed among the new generation of Chinese immigrants in Italy. Through the lens of linguistic capital theory, this phenomenon essentially represents a reconfiguration of value within the specific “linguistic market” inhabited by the new generation.

For the new generation, who are still in their schooling years, mastering advanced Chinese literacy requires a substantial investment of extracurricular time. However, within the Italian mainstream education system and peer social fields—upon which their survival and development depend—this advanced literacy cannot be directly converted into acceptance by the mainstream group or academic achievement; thus, its “exchange value” remains relatively limited. Consequently, the new generation effects a strategic “functional displacement” in their language practices: they no longer pursue Chinese as a form of “skill capital” requiring precise mastery (i.e., grammatical correctness and fluency in reading and writing), but rather transform it into a form of “symbolic capital” emphasizing the legitimacy of identity. In this reconfigured value system, the “legitimacy” of language is no longer determined by adherence to orthographic norms, but by its recognizability within the Chinese community. As long as an individual can maintain basic oral interaction or sustain an affective attachment to the language at a psychological level, Chinese suffices to fulfill its symbolic function as a “cultural credential.” This mechanism explains how the new generation, while circumventing their deficits in literacy skills, manages to secure a sense of safety and confirmation regarding their identity through symbolic operations.

### 4.2 Identity performance in the peer domain

By examining data on language use across different interlocutors and settings, this study identifies the peer domain as the critical arena for the enactment of the symbolic function of language. The findings indicate that while Italian dominates peer interactions among the new generation, Chinese has not completely exited the stage; instead, it persists in a form characterized by fragmentary code-switching.

This linguistic pattern possesses the distinct quality of “identity performance.” The theory of “Acts of Identity” proposed by Le Page and Tabouret-Keller (1985) suggests that an individual’s linguistic behavior is fundamentally a projection, wherein one declares affiliation to a specific social group through the selection of particular linguistic forms. In peer interactions, the occasional interspersion of Chinese vocabulary or short phrases by the new generation is not primarily intended to enhance communicative efficiency. Rather, it serves to demarcate an invisible internal group boundary within the public space. Through this specific linguistic behavior, the new generation of Chinese immigrants proclaims their distinct identity vis-à-vis the mainstream group while simultaneously reinforcing the bonds among members within the group. This symbolic linguistic practice requires neither high-level complex expression nor the support of advanced writing skills; it relies instead on a tacit understanding of shared cultural codes among peers. Therefore, even when literacy is constrained, Chinese effectively maintains group cohesion through these micro-level social interactions.

### 4.3 Alternative pathways for identity maintenance

The structural decoupling revealed in this study essentially represents a specialized, adaptive identity maintenance mechanism formed by the new generation under ecological pressure—an identity pathway that lies behind the symbolization of language function and remains independent of literacy skills. Within this pathway, daily family interactions, peer identity performances, and affective expressions of Chinese culture have become the core elements for sustaining identity, while the high-difficulty task of character literacy has been marginalized as a non-essential auxiliary skill. This mechanism not only ensures the stability of ethnic identity during intergenerational transmission but also explains why the low-proficiency group, despite lacking the support of written language capabilities, maintains a level of identity comparable to that of the high-proficiency group. Furthermore, this demonstrates that in specific immigrant contexts, the persistence of identity possesses remarkable resilience, capable of transcending the limitations of linguistic proficiency to exist in its own right.

## 5 Conclusion and recommendations

### 5.1 Research conclusions

This study confirms that although the Chinese literacy proficiency of the new generation of Chinese immigrants in Italy remains relatively weak, their sense of identity has not diminished concurrently. The coexistence of “low literacy and high identity” reveals a substantive shift in the function of the Chinese language: it has evolved from a mere instrument prioritizing communicative efficiency into a symbolic marker establishing ethnic affect and boundaries. Further analysis indicates that, relying on oral interactions within the family and identity performances among peers, the new generation has constructed a logic of identity that functions independently of advanced literacy skills. This de-skilled mode of symbolic usage enables identity to maintain a high degree of resilience and stability despite the reality of relatively weak literacy proficiency.

On a theoretical level, this study makes specific micro-level contributions to existing frameworks of language and identity. Previous studies exploring the symbolic function of language have often lacked in-depth analysis of how Italian–Chinese youth concretely implement these symbolic practices in authentic multilingual interactions. Through a mixed-methods approach, this study successfully links macro-level symbolic capital theory with micro-level daily interaction data and subdivided language proficiency indicators. The findings demonstrate that within the specific European multilingual ecology, micro-level symbolic linguistic behaviors in daily communication substantively fill the identity vacuum that might otherwise result from weakened literacy skills.

### 5.2 Research recommendations

At the family level, parents should establish the principle that prioritizing the protection of symbolic identity supersedes the enforcement of skill attainment. Research demonstrates that the sense of identity based on symbolic functions serves as the psychological cornerstone for maintaining Chinese heritage. In family language planning, high-pressure strategies stemming from excessive anxiety over literacy outcomes must be avoided; such approaches risk undermining the child’s existing cultural confidence and may even precipitate a rebellious psychological stance toward their identity. Parents should, on the basis of maintaining this innate identification, protect the child’s interest in learning by fostering a relaxed linguistic atmosphere, thereby reserving “psychological space” for subsequent advancement in literacy.

At the level of Chinese language education, instructional design should adeptly utilize the “symbolic function” as a scaffold for literacy pedagogy. Leveraging this psychological mechanism, curricula for younger or entry-level students should incorporate culturally symbolic courses to prioritize the satisfaction of their needs for identity expression. Only after students have acquired a sufficient sense of achievement and belonging at the symbolic level should normative literacy training be introduced progressively. This pathway of “symbol first, skill later” facilitates a natural transition from the symbolic need of “confirming identity” to the technical need of “mastering the tool,” thereby enhancing language proficiency without compromising the sense of identity.

### 5.3 Research limitations

While confirming the findings of this study, it is necessary to acknowledge objective limitations regarding both sampling representativeness and scale validation. First, in terms of sampling, all respondents were recruited from physical Chinese heritage schools in Italy. This approach introduces a certain systematic bias, as students attending heritage schools and their families largely already possess positive attitudes toward the Chinese language and culture. Consequently, the sample fails to encompass unaffiliated Chinese youth, fully assimilated individuals, or those who have never participated in any form of heritage language education. This implies that the findings might, to some extent, overestimate the overall strength of identity and the symbolic attachment to Chinese within the broader demographic. Second, regarding research instruments and data validation, due to constraints in objective data processing conditions and sample size, this study primarily relied on descriptive statistical analysis and the triangulation of qualitative interview data to support its conclusions. It did not employ complex psychometric methods for reliability testing (such as calculating Cronbach’s alpha), nor did it report rigorous Confirmatory Factor Analysis (CFA) data. Although the questionnaire items were rigorously constructed based on established heritage language theories, the lack of a quantitative construct validity validation model remains a methodological limitation. Future research should, on the one hand, attempt to broaden the sampling scope to include participants from more diverse backgrounds; on the other hand, with a larger sample size, future studies could introduce advanced statistical validation tools, such as structural equation modeling, to conduct a deeper quantitative examination of the scale’s internal structure.

## Data Availability

The original contributions presented in the study are included in the article/supplementary material, further inquiries can be directed to the corresponding author.

## References

[ref9001] Adler A. (1927). Understanding Human Nature. New York: Greenberg.

[ref9002] Ang I. (2001). On not Speaking Chinese: Living between Asia and the West. London: Routledge.

[ref9003] Bai S. Qin L. Yang L. (2014). Multidisciplinary interpretation and interdisciplinary integration of the concept of identity. Academics 11, 80–90. doi: 10.3969/j.issn.1002-1698.2014.11.008

[ref9004] Baker C. (1992). Attitudes and Language. Clevedon: Multilingual Matters.

[ref9005] Bao R. Li B. (2023). Research on cultural identity and influencing factors of Chinese-origin youth: a case study of campers in "root-seeking tour". Int. Chin. Lang. Educ. Res. 1, 1–22. doi: 10.12677/ap.2024.147470

[ref6] Bourdieu P. (1991). Language and Symbolic Power. Cambridge: Polity Press.

[ref7] Cao X. (2014). A re-examination of overseas Chinese teaching from the perspective of “heritage language” theory. J. Chin. Lang. Teach. Res. 4, 48–56. doi: 10.16131/j.cnki.cn44-1669/g4.2014.04.006

[ref9008] Cao Y. Yang Y. (2014). A review of Western research on the relationship among language, culture, and identity in the last twenty years. J. Kunming Univ. Scie. Technol. (Soc. Sci. Edn.) 14, 95–99. doi: 10.16112/j.cnki.53-1160/c.2014.05.029

[ref9010] Edwards J. (1985). Language, Society and Identity. Oxford,UK: Basil Blackwell.

[ref9011] Erikson E. H. (1950). Childhood and Society. New York: W. W. Norton & Company.

[ref9012] Ferrer R. C. Sankoff D. (2003). Identity as the primary determinant of language choice in Valencia. J. Sociolinguist. 7, 50–64. doi: 10.1111/1467-9481.00211

[ref9013] Giles H. Johnson P. (1987). Ethnolinguistic identity theory: a social psychological approach to language maintenance. Int. J. Sociol. Lang. Berlin,Germany, 1987, 69–100. doi: 10.1515/ijsl.1987.68.69, 31755547

[ref14] Guo X. (2015). On the three major divisions of Chinese language teaching. Stud. Chin. Lang. Stud. Chin. Lang. 5, 475–478.

[ref15] Guo X. (2017). On ancestral language and ancestral language inheritance. Lang. Strategy Res. 2, 10–19. doi: 10.19689/j.cnki.cn10-1361/h.2017.03.005

[ref16] Guo X. (2023). Constructing the discourse system of overseas Chinese heritage language. Appl. Linguis. 2, 2–10. doi: 10.16499/j.cnki.1003-5397.2023.02.007

[ref17] He A. W. (2006). Toward an identity theory of the development of Chinese as a heritage language. Herit. Lang. J. 4, 1–28. doi: 10.46538/hlj.4.1.1

[ref18] He H. (2023). Research on heritage language use and identity of the second generation of Chinese in Singapore. J. Yunnan Norm. Univ. (Teach. Res. Chin. For. Lang. Edn.) 21, 42–52. doi: 10.16802/j.cnki.ynsddw.2023.01.007

[ref19] King K. A. Fogle L. Logan-Terry A. (2008). Family language policy. Lang. Linguist. Compass 2, 907–922. doi: 10.1111/j.1749-818X.2008.00076.x

[ref20] Labov W. (1966). The Social Stratification of English in New York City. Washington: Center for Applied Linguistics.

[ref9014] Lay C. Verkuyten M. (1999). Ethnic identity and its relation to personal self-esteem: a comparison of Canadian-born and foreign-born Chinese adolescents. J. Soc. Psychol. 139, 288–299. doi: 10.1080/00224549909598385, 10410617

[ref22] Le Page R. B. Tabouret-Keller A. (1985). Acts of Identity: Creole-based Approaches to Ethnicity and Language. Cambridge: Cambridge University Press.

[ref23] Paciocco A. (2018). Performing Chinese diasporic identity through mandarin: the case of Italian-schooled Chinese migrant youth in Prato (Italy). J. Lang. Identity Educ. 17, 207–221. doi: 10.1080/15348458.2018.1437348

[ref24] Paciocco A. E. (2021). Chinese maintenance and shift among Chinese migrant youth in Prato (Italy) and its connectedness with new formations of Chinese identity. Global Chin. 7, 29–55. doi: 10.1515/glochi-2021-0002

[ref25] Rajagopalan K. (2016). Language and identity: national, ethnic, religious. Word 62, 276–280. doi: 10.1080/00437956.2016.1248656

[ref9015] Shen L. (2017). A comparative study of cultural identity of the new generation of overseas Chinese in Southeast Asia by country. J. Res. Ethnic Educ. 28, 124–129. doi: 10.15946/j.cnki.1001-7178.2017.06.020

[ref9016] Smolicz J. J. (1981). Core values and cultural identity. Ethnic Racial Stud. 4, 75–90. doi: 10.1080/01419870.1981.9993325

[ref28] Stratilaki S. (2012). Plurilingualism, linguistic representations and multiple identities: crossing the frontiers. Int. J. Multiling. 9, 189–201. doi: 10.1080/14790718.2011.644559

[ref29] Tajfel H. (1978). Differentiation Between Social Groups: Studies in the Social Psychology of Intergroup Relations. London: Academic Press.

[ref30] Wang A. (2004). A case study on language and identity of Chinese-Indonesian youth: an investigation of Chinese-Indonesian students at the College of Chinese Language and Culture, Huaqiao University. Overseas Chin. Hist. Stud. 4, 23–31. doi: 10.3969/j.issn.1002-5162.2004.04.004

[ref31] Wang A. (2006). Chinese language use and Chinese identity: an investigation of over 400 Chinese-Indonesian students. J. Fuzhou Univ. (Philos. Soc. Sci. Edn.) 4, 86–90. doi: 10.3969/j.issn.1002-3321.2006.04.017

[ref9017] Wang J. Yan X. (2011). Investigation on cultural identity of Chinese-origin youth in Wenzhou. Bagui Overseas Chin. J. 1, 24–27.

[ref33] Xu X. (2022). A study on the identity of 136 Chinese American high school students and its impact on their Chinese learning motivation. [Master's thesis, East China Normal University].

[ref9018] Zhang H. (2015). New developments in empirical research on overseas language and identity. For. Lang. Res. 3, 42–46. doi: 10.13978/j.cnki.wyyj.2015.03.009

[ref9019] Zhang H. (2020). Investigation and research on cultural identity of Chinese-origin students in Madagascar. [Master's thesis, Shandong Normal University]

[ref9020] Zhang Q. Wang H. Zhang J. (2021). A study on ancestral language maintenance of the second generation of Chinese in Italy. J. Chin. Lang. Teach. Res. 2, 61–70. doi: 10.16131/j.cnki.cn44-1669/g4.2021.02.008

[ref37] Zhou M. (2014). Language identity and Chinese heritage language education. J. Chin. Lang. Teach. Res. 1, 15–20. doi: 10.16131/j.cnki.cn44-1669/g4.2014.01.018

[ref38] Zhou Q. (2016). A review of domestic research on language and identity. Lang. Strategy Res. 1, 72–79. doi: 10.19689/j.cnki.cn10-1361/h.2016.01.010

[ref39] Zhu W. Wang J. (2012). A comparative study on identity of cross-cultural ethnic groups and Chinese communication strategies. J. Yunnan Norm. Univ. (Teach. Res. Chin. For. Lang. Edn.) 10, 65–71. doi: 10.16802/j.cnki.ynsddw.2012.03.010

